# Design of High-Performance Polybenzoxazines with Tunable Extended Networks Based on Resveratrol and Allyl Functional Benzoxazine

**DOI:** 10.3390/polym12122794

**Published:** 2020-11-26

**Authors:** Yunliang Xing, Xianru He, Rui Yang, Kan Zhang, Shengfu Yang

**Affiliations:** 1 School of New Energy and Materials, Southwest Petroleum University, Chengdu 610500, China; Phoebe@coryes.com; 2 Research School of Polymeric Materials, School of Materials Science and Engineering, Jiangsu University, Zhenjiang 212013, China; yr183346@163.com; 3 Department of Chemistry, University of Leicester, Leicester LE1 7RH, UK; sfy1@le.ac.uk

**Keywords:** benzoxazine, resveratrol, polybenzoxazine, allyl, high-performance

## Abstract

A novel resveratrol-based bio-benzoxazine monomer (RES-al) containing an allyl group has been synthesized using resveratrol, allylamine, and paraformaldehyde via Mannich condensation reaction, and its chemical structures have been characterized by FT-IR spectroscopy and NMR techniques. The polymerization behavior of this benzoxazine resin has been investigated using in situ FT-IR and differential scanning calorimeter (DSC) measurements, and the thermal-mechanical properties of its corresponding polybenzoxazines are evaluated by DMA and TGA. We show that by controlling the curing process of the oxazine ring, the C=C bond in resveratrol, and the allyl group in RES-al, the cross-linking network of the polybenzoxazine can be manipulated, giving rise to tunable performance of thermosets. As all curable functionalities in RES-al are polymerized, the resulted polybenzoxazine exhibits a good thermal stability with a *Tg* temperature of 313 °C, a T_d5_ value of 352 °C, and char yield of 53% at 800 °C under N_2_.

## 1. Introduction

Benzoxazines are new types of well-commercialized thermosetting resins, which are generally prepared from phenolic derivatives, primary amines, and formaldehyde/paraformaldehyde based on Mannich condensation reaction [1,2,3,4]. Benzoxazine (BZ) resins have been found to possess physical characteristics superior to those of most traditional phenolic or epoxy thermosets attributed to their cross-linked structures with various hydrogen bonds after thermal curing [5]. Besides, benzoxazine resins could be simply thermally activated polymerized via a cationic polymerization with/without initiators [1,2]. Polybenzoxazines (PBZs), as the polymerized products of benzoxazine resins, possess many attractive properties that are important for various applications, including superb chemical resistance [6], high thermal stability [7,8,9], excellent mechanical properties [10,11], and low dielectric constants [12,13,14]. Recently, polybenzoxazine thermosets based on tri-oxazine ring functional benzoxazines have demonstrated much higher thermal stability than the thermoset obtained from the well-commercialized bisbenzoxazine monomers [15,16,17,18,19,20,21]. Specifically, the addition of greater number of oxazine-ring functionalities in monomer provides a useful means to further improve the comprehensive properties of thermosets [15].

Resveratrol has been widely investigated, since it was isolated from white hellebore in 1940 [22,23,24,25]. The phenolic hydroxyl groups in resveratrol make it a good candidate for developing various thermosets [17,18]. For instance, resveratrol-based epoxy, cyanate ester, and benzoxazine thermosetting resins have been extensively reported, and all of those resveratrol-derived thermosets show high thermomechanical properties, indicating them as promising alternatives to petroleum-based thermosetting systems [22,26].

The present work reports a new resveratrol-derived tri-oxazine functional benzoxazine monomer containing an allyl group. The ring-opening polymerization behavior of the obtained benzoxazine monomer was investigated. In addition, the effects of resveratrol and allyl groups on thermal properties of the derived benzoxazine have been evaluated.

## 2. Experimental Section

### 2.1. Materials

Resveratrol (99%) and allylamine were purchased from Aladdin Reagent, Shanghai, China. Paraformaldehyde (99%), sodium hydroxide (NaOH), and toluene were obtained from Energy Reagent, Shanghai, China.

### 2.2. Characterization

^1^H, ^13^C, ^1^H–^1^H NOESY and ^1^H–^13^C HMQC NMR spectra were obtained on a Bruker AVANCE II 400 MHz (Bruker, Fällanden, Switzerland) spectrometer in DMSO-d_6_. Tetramethyl silane was used as an internal standard. FT-IR spectrophotometer (Nicolet Nexus 670, Nicolet, WI, USA) was carried out to further confirm the existence of each functionality in benzoxazine. Each spectrum was obtained in a resolution of 4 cm^−1^, and the frequency range for the scans was set from 4000 to 400 cm^−1^. Elementary analyzer (Elementar Vario EL-III, Hanau, Germany) was also adopted to confirm the chemical structure. A NETZSCH differential scanning calorimeter (DSC) (Model 204f1, NETZSCH, Selb, Germany) was performed to obtain DSC thermogram of each sample. The temperature ramp rate was set as 10 °C/min in N_2_ (a N_2_ flow rate of 60 mL/min). In addition, dynamic mechanical analysis (DMA) was performed by a NETZSCH DMA/242E analyzer (NETZSCH, Selb, Germany) at a heating rate of 3 °C/min. Thermal gravimetric analysis (TGA) was performed on a NETZSCH STA449-C thermogravimetric analyzer (NETZSCH, Selb, Germany) under both N_2_ and air atmospheres at a flow rate of 40 mL/min. Moreover, the heating ramp rate for TGA was set as 10 °C/min.

### 2.3. Synthesis of Tri-Functional Benzoxazine (RES-al)

Into a 100 mL flask were mixed resveratrol (1.14 g, 5 mmol), allylamine (0.86 g, 15 mmol), paraformaldehyde (0.99 g, 33 mmol), and 35 mL of toluene. The mixture was stirred at 100 °C for 6 h. Then, the mixture was cooled down, and the organic layer in solution was washed by 2 N NaOH solution for 3 times. Afterwards the solution was further washed by distilled water. After removing the solvent, the crude product was purified through recrystallization in acetone/toluene mixtures (1:1). At last, white crystals were obtained (yield ca. 82%). ^1^H NMR (400 MHz, CDCl_3_), ppm: *δ* = 3.36–3.44 (m, 6H, N–C*H*_2_–CH=), 3.93, 4.04 (d, 6H, Ar–C*H*_2_–N, oxazine), 4.84, 4.85, 4.91 (t, 6H, O–C*H*_2_–N, oxazine), 5.21–5.30 (m, 6H, –CH=C*H*_2_), 5.88–6.00 (m, 3H, –C*H*=CH_2_), 6.68 (s, 1H, Ar), 6.82 (d, 1H, CH=C*H*–Ar), 6.86 (d, 1H, Ar), 7.08 (d, 1H, Ar–C*H*=CH), 7.16–7.30 (3H, Ar). FT-IR spectra (KBr), cm^−1^: 3077 (=C–H stretching vibration of allyl), 1605 (C=C stretching in resveratrol), 1236 (C–O–C asymmetric stretching), 930 (benzoxazine related band). Anal. Calcd. for C_29_H_33_N_3_O_3_: C, 73.86; H, 7.05; N, 8.91. Found: C, 73.81; H, 7.10; N, 8.87.

### 2.4. Polymerization Procedures of RES-al 

Polybenzoxazines were obtained using different stepwise polymerization procedures via RES-al. RES-al was first cured in a mold by multiple curing steps of 1 h each at 140, 180, and 220 °C to produce a partially polymerized sample, namely, poly(RES-al)-1. Heating treatment of poly(RES-al)-1 at 260 °C for 1 h in the same mold generated poly(RES-al)-2, the further heating of which at 300 °C for 1 h gave rise to a fully cured thermoset, poly(RES-al)-3.

## 3. Results and Discussion

### 3.1. Synthesis and Structural Confirmation of RES-al

**Scheme 1:**
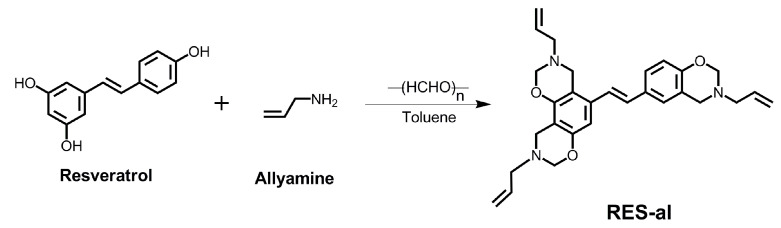
Synthesis of the benzoxazine monomer (RES-al).

**Figure 1:**
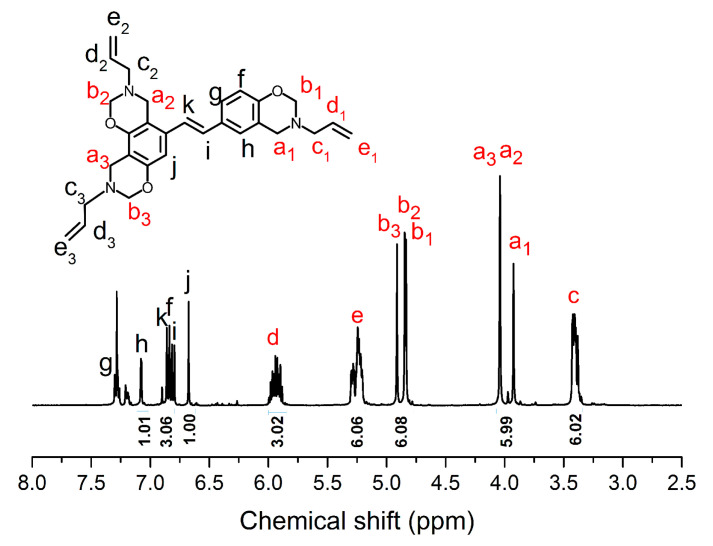
^1^H NMR spectrum of RES-al in CDCl_3_.

The resveratrol-derived tri-oxazine containing benzoxazine resin, RES-al, was successfully obtained by a modified Mannich condensation from resveratrol, allylamine, and paraformaldehyde (Scheme 1). The NMR proton and carbon assignments for the oxazine ring and the allyl group in RES-al are determined by both 2D ^1^H-^1^H NOESY and ^1^H-^13^C HMQC NMR spectra (Figures S1 and S2). As can be seen in Figure 1, the characteristic proton resonances in Ar–C*H*_2_–N group from the oxazine ring of RES-al show overlapped doublets at 3.93 and 4.04 ppm. Besides, the triplet signals for the O–C*H*_2_–N group can be observed at 4.84, 4.85, and 4.91 ppm, respectively; the characteristic proton resonances of the allyl group in RES-al can be observed around 3.4, 5.2, and 5.9 ppm, respectively [27].

**Figure 2:**
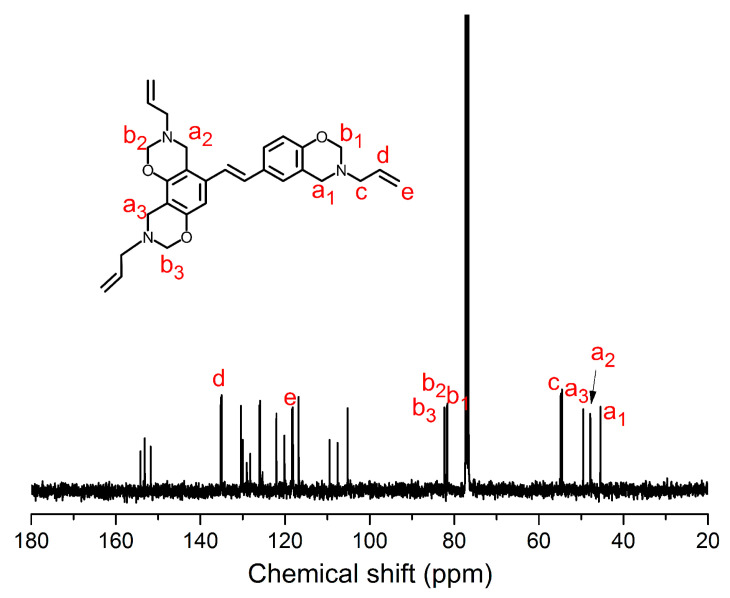
^13^C NMR spectrum of RES-al in CDCl_3_.

Figure 2 shows the ^13^C NMR spectrum, which was provided to confirm the chemical structure of this monomer. As can be observed in Figure 2, two sets of triplet signals at the range of 45–50 and 81–83 ppm are characteristic carbon resonances for Ar–*C*H_2_–N– and –O–*C*H_2_–N–, respectively [4]. In addition, other typical carbon resonances of allyl group in RES-al can also be found around 54, 118, and 135 ppm, respectively [17]. Therefore, the above NMR results fully support that we have successfully obtained the target benzoxazine compound.

**Figure 3:**
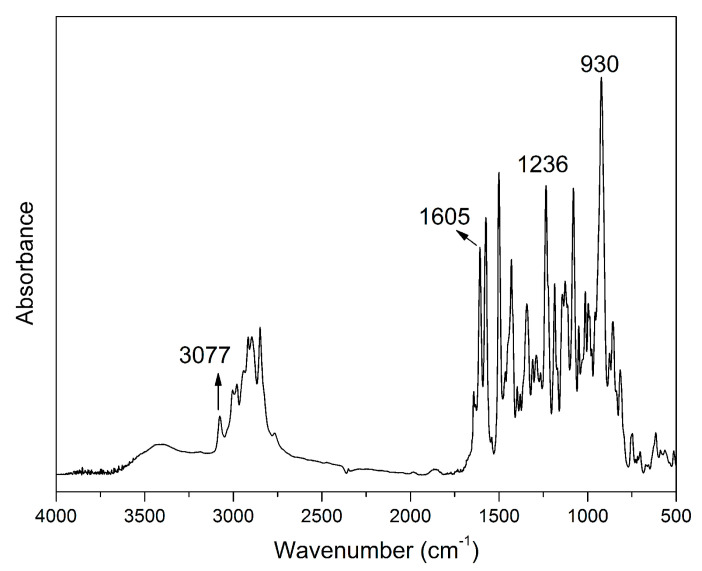
FT-IR spectra of RES-al.

FT-IR spectrum was obtained in this study to further confirm the existence of each functionality in this allyl-containing benzoxazine derived from resveratrol. As depicted in Figure 3, the band at 3077 cm^−1^ is attributed to the C–H stretching vibration in allyl group. In addition, the characteristic bands at around 1605 cm^−1^ are from the C=C stretching of resveratrol in benzoxazine structure. Moreover, the presence of the oxazine ring aromatic ether is evidenced by characteristic peak (C–O–C antisymmetric stretching) at 1236 cm^−1^ [28]. Furthermore, the typical band at 930 cm^−1^ is related to the oxazine ring [29].

### 3.2. Polymerization Behaviors of the Benzoxazine Monomer

**Figure 4:**
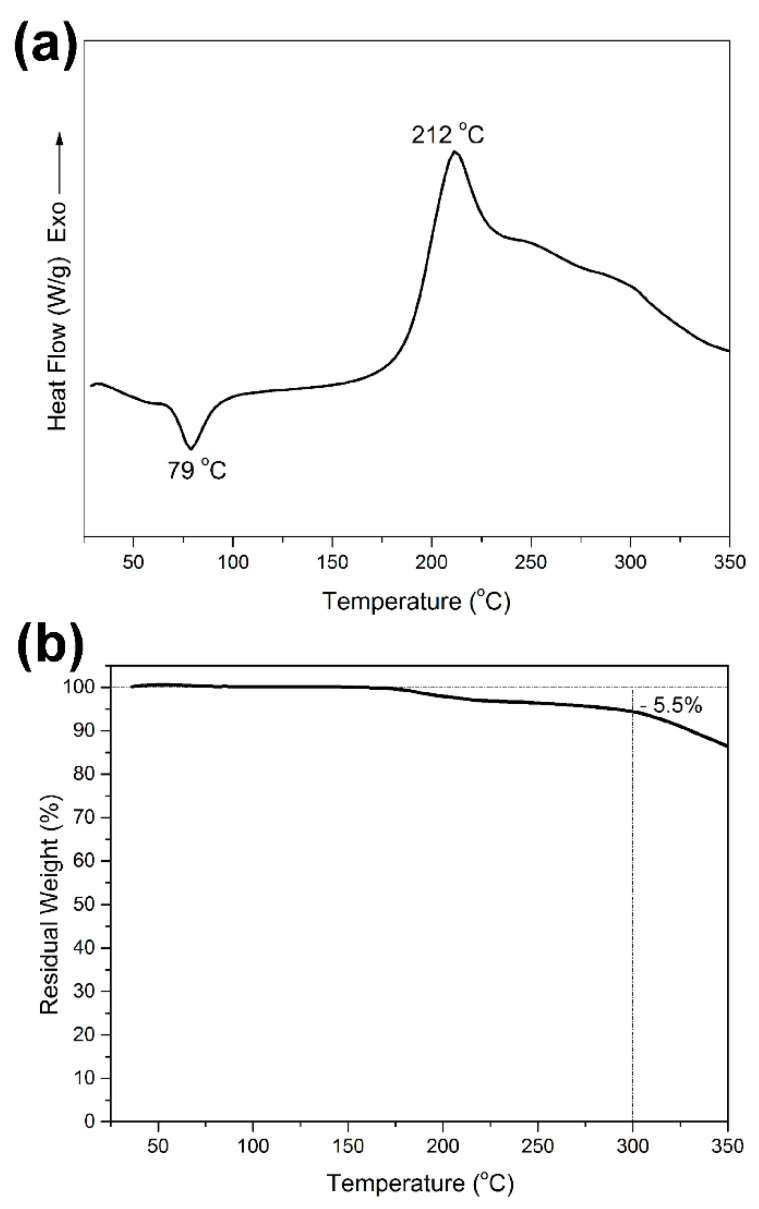
Differential scanning calorimeter DSC (**a**) and TGA (**b**) thermograms of RES-al.

The polymerization behavior during the thermal treatment is shown by the DSC thermograms of RES-al in Figure 4a. An endothermic peak at 79 °C suggests the good purity of this allyl-containing resveratrol-based benzoxazine monomer. RES-al exhibits a very broad exothermic peak with a maximum at 212 °C, which is very different from other well-known mono- or bis-benzoxazine resins [30,31,32]. The exothermic peak consists of three overlapped peaks, including the ring-opening polymerization, and other two reactions of resveratrol and allyl groups in benzoxazine. The three oxazine rings in the same molecule are expected to alter the curing reaction due to the fully asymmetric structure of RES-al. Besides, the curing process of the C=C bond in allyl group could also extend the polymerization behavior of RES-al. Figure 4b shows the TGA thermogram of RES-al. A 5.5% of weight loss can be found before 300 °C for RES-al, which is much lower than the resveratrol and aniline based tri-benzoxazine resin. This suggests that the additional cross-linkage from the allyl group in RES-al helps to reduce the degradation during the curing processes. However, it is difficult to calculate the enthalpy of the polymerization of each reaction due to the overlapped polymerization behaviors involved during the polymerization of RES-al. Moreover, the additional degradation behavior also affects the enthalpy of the polymerization.

**Figure 5:**
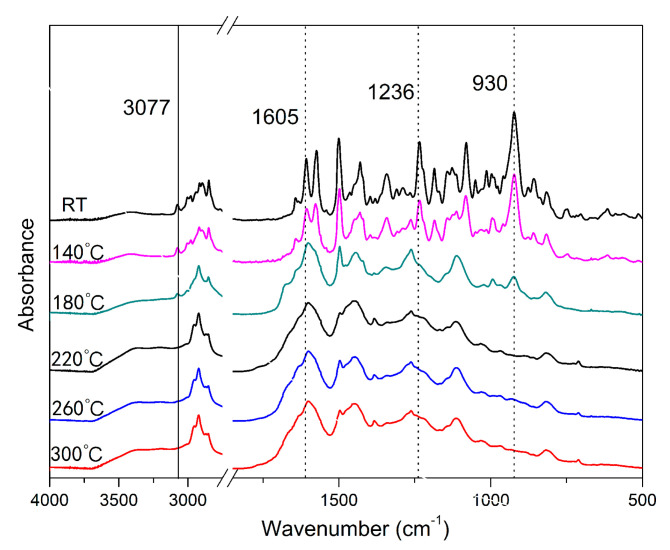
In situ FT-IR spectra of RES-al after various thermal treatments.

To systematically investigate the polymerization behaviors of this newly obtained benzoxazine resin, in situ FT-IR analyses were then employed, which utilized the typical band at 1236 cm^−1^ (C–O–C asymmetric stretching) and 930 cm^−1^ (oxazine ring related mode) to analyze the ring-opening polymerization behavior of RES-al (see Figure 5). The phenolic hydroxyl band in the region of 3700–3200 cm^−1^ obviously increases at higher temperature. Meantime, both the characteristic C–H stretching vibration of the substituted allyl group at 3077 cm^−1^ and the C=C stretching of resveratrol at 1605 cm^−1^ decrease after heating, and completely disappear at 300 °C. These observations suggest that the exothermic peak from the above DSC curve of RES-al involves three types of curing routes, including the oxazine ring polymerization, the olefin polymerization through the double bonds from resveratrol, and from the allyl functionalities.

**Figure 6:**
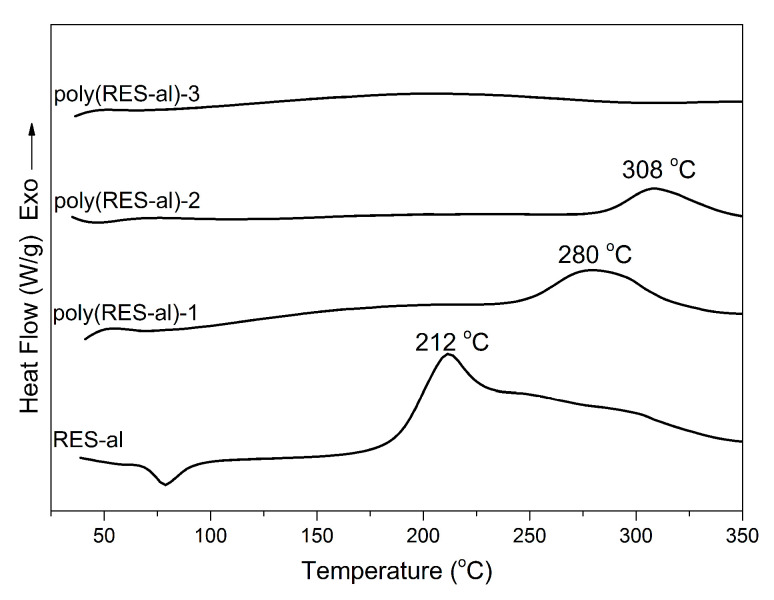
DSC thermograms of RES-al and its related thermosets recorded under N_2_ at a heating rate of 10 °C/min.

**Scheme 2:**
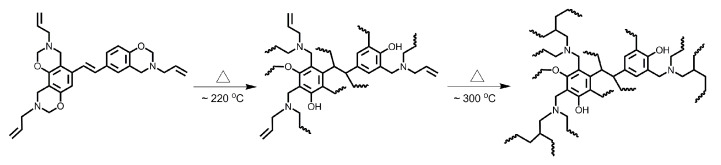
Schematic representation of proposed thermally activated polymerization processes of RES-al.

DSC was also applied to investigate the thermal behaviors of RES-al based thermosets. Figure 6 displays DSC thermograms of RES-al and its related thermosets. Clearly, the exothermic peak gradually decreased in intensity after the further heating treatment and fully disappeared for poly(RES-al)-3, suggesting the completion of the thermally activated polymerization behaviors in RES-al. In addition, the exothermic peak at 280 and 308 °C of poly(RES)-al-1 and poly(RES-al)-2 may represent the reactions of resveratrol and allyl groups after the polymerization of oxazine ring. In general, it is considered that there are two reaction approaches involved in the polymerization of benzoxazine. The first step is the ring-opening polymerization and followed with the electrophilic substitution. The final polybenzoxazine may have phenolic structure, phenoxyl structure, or both [4]. As the reaction site on the aromatic ring are highly substituted in the left part of the molecule in RES-al, the oxazine ring in the left part could be polymerized to form phenoxyl structure. Therefore, we finally give a proposed polymerization behavior of RES-al as shown in Scheme 2.

### 3.3. Thermal Properties of Thermosets Derived from RES-al

**Figure 7:**
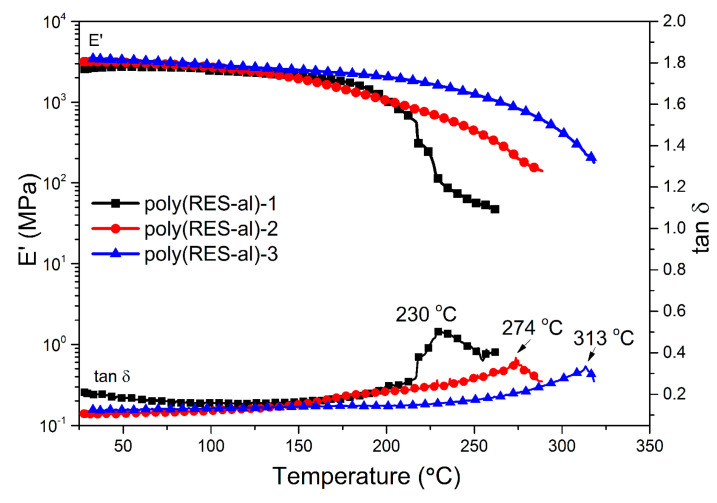
Dynamic mechanical spectra of polybenzoxazines.

The thermomechanical performances of RES-al derived thermosets were evaluated by DMA, and the glass transition temperature (*T*_g_) of each thermoset was determined by the maximal of the tan δ peak. As depicted in Figure 7, poly(RES-al)-1, poly(RES-al)-2, and poly(RES-al)-3 show *T*_g_ temperatures at 230, 274, and 313 °C, respectively. In all of the cases, the *T*_g_ values increase with the polymerization temperature and are all higher than their corresponding polymerization temperatures. Poly(RES-al)-3 exhibits higher storage modulus value than both poly(RES-al)-1 and poly(RES-al)-2 from room temperature to 300 °C. The excess crosslink points attributed from the C=C in allyl and resveratrol significantly increase the overall cross-linking density, which increases the *T*_g_ of RES-al based thermosets.

**Figure 8:**
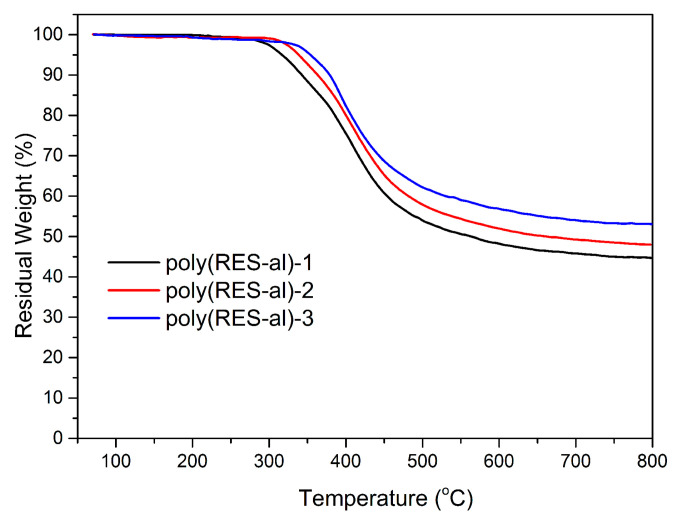
Thermogravimetric analysis of RES-al based thermosets in N_2_.

**Figure 9:**
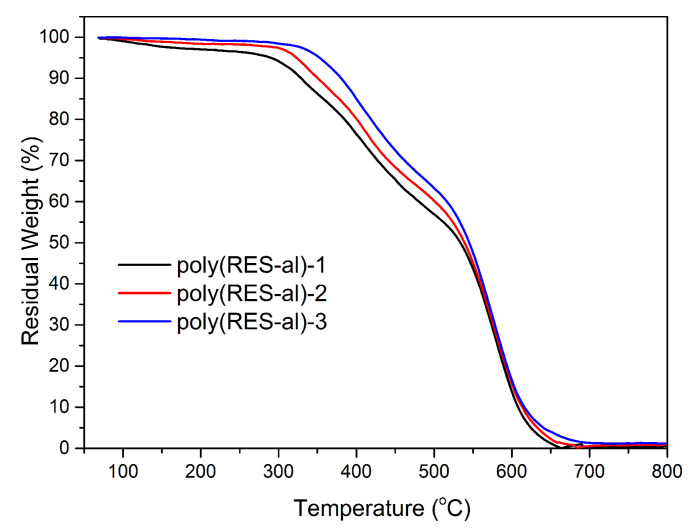
Thermogravimetric analysis of RES-al based thermosets in air.

The thermal stabilities of RES-al based thermosets were evaluated by TGA under both N_2_ and air conditions (see Figure 8 and Figure 9). Notably, the temperatures of T_d5_ and T_d10_ of these thermosets are much higher than some other reported thermosets generated from traditional benzoxazine resins [33,34]. Herein, some initial weight loss in both nitrogen and air can be observed for poly(RES-al)-1 and poly(RES-al)-2, which is because of the decomposition of defect structures in their cross-lined networks [35], but poly(RES-al)-3 showed even better thermal stability, with the highest T_d5_ of 352 °C (in both N_2_ and air), and T_d10_ of 378 °C in N_2_ and 380 °C in air. The improved thermal stability of thermoset is mainly attributed to the multiple cross-linked polymer structure generated from the oxazine ring-opening polymerization and the additional polymerization of the C=C groups in resveratrol and allyl groups, which also significantly reduces the defects in terminal chain-ends. Poly(RES-al)-3 also exhibits a high char yield value of 53% at 800 °C in N_2_. All data related to thermal properties of thermosets are summarized in Table 1. We should also point out that the enhanced thermal stability of poly(RES-al) is also partially attributed to the removing of some defects in the polybenzoxazine network during the high temperature polymerization. As a result, the excellent thermal properties suggest that resveratrol-based thermosets reported here are promising materials for high-temperature applications.

## 4. Conclusions

A new tri-benzoxazine resin based on resveratrol and allyl groups was synthesized and characterized in the current work. The thermally activated polymerization of RES-al proceeded in multiple curing approaches, including the ring-opening polymerization of oxazine ring and the polymerization of C=C bonds in both resveratrol and allyl groups in benzoxazine. Notably, the resulting fully polymerized thermoset showed prominent thermal stability with high T_d5_ (352 °C) and T_d10_ (378~380 °C) values in both N_2_ and air. This newly obtained thermoset also exhibited high *T*_g_ of 313 °C and a high char yield value of 53% at 800 °C under nitrogen atmosphere. The excellent thermal properties suggest the great potential of the thermosets derived from this newly obtained resveratrol and allyl-based tri-benzoxazine resin in high-performance areas.

## Figures and Tables

**Table 1:** Thermal properties of RES-al based thermosets.

Sample	*T*_g_ (DMA)		N_2_		Air	
	(°C)	T_d5_ (°C)	T_d10_ (°C)	Yc	T_d5_ (°C)	T_d10_ (°C)
poly(RES-al)-1	230	318	342	45	290	328
poly(RES-al)-2	274	338	363	48	321	349
poly(RES-al)-3	313	352	378	53	352	380

## Supplementary Materials

The following are available online at https://www.mdpi.com/2073-4360/12/12/2794/s1, Figure S1: ^1^H-^1^HNOESY 2D NMR spectrum of RES-al, Figure S2: ^1^H-13C HMQC 2D NMR spectrum of RES-al.

## References

[1] Ning X., Ishida H. (1994). Phenolic materials via ring-opening polymerization: Synthesis and characterization of bisphenol-A based benzoxazines and their polymers. J. Polym. Sci. Part A Polym. Chem..

[2] Ghosh N.N., Kiskan B., Yagci Y. (2007). Polybenzoxazines—New high performance thermosetting resins: Synthesis and properties. Prog. Polym. Sci..

[3] Ishida H., Froimowicz P. (2017). Advanced and Emerging Polybenzoxazine Science and Technology.

[4] Ishida H., Agag T. (2011). Handbook of Benzoxazine Resins.

[5] Shen X., Cao L., Liu Y., Dai J., Liu X., Zhu J., Du S. (2018). How does the hydrogen bonding interaction influence the properties of polybenzoxazine? An experimental study combined with computer simulation. Macromolecules.

[6] Ohashi S., Kilbane J., Heyl T., Ishida H. (2015). Synthesis and characterization of cyanate ester functional benzoxazine and its polymer. Macromolecules.

[7] Liu J., Safronava N., Lyon R.E., Maia J., Ishida H. (2018). Enhanced thermal property and flame retardancy via intramolecular 5-membered ring hydrogen bond-forming amide functional benzoxazine resins. Macromolecules.

[8] Kolanadiyil S.N., Bijwe J., Varma I.K. (2013). Synthesis of itaconimide/nadimide-functionalized benzoxazine monomers: Structural and thermal characterization. React. Funct. Polym..

[9] Yang P., Gu Y. (2012). Synthesis of a novel benzoxazine-containing benzoxazole structure and its high performance thermoset. J. Appl. Polym. Sci..

[10] Chaisuwan T., Ishida H. (2006). High-performance maleimide and nitrile-functionalized benzoxazines with good processibility for advanced composites applications. J. Appl. Polym. Sci..

[11] Zhang K., Yu X. (2018). Catalyst-free and low-temperature terpolymerization in a single-component benzoxazine resin containing both norbornene and acetylene functionalities. Macromolecules.

[12] Chen S., Ren D., Li B., Li K., Chen L., Xu M., Liu X. (2019). Benzoxazine containing fluorinated aromatic ether nitrile linkage: Preparation, curing kinetics and dielectric properties. Polymers.

[13] Wu J., Xi Y., Mccandless G.T., Xie Y., Menon R., Patel Y., Menon R., Patel Y., Yang D.J., Iacono S.T. (2015). Synthesis and characterization of partially fluorinated polybenzoxazine resins utilizing octafluorocyclopentene as a versatile building block. Macromolecules.

[14] Zhang K., Yu X., Kuo S.W. (2019). Outstanding dielectric and thermal properties of main chain-type poly(benzoxazine-co-imide-co-siloxane)-based cross-linked networks. Polym. Chem..

[15] Sini N.K., Endo T. (2016). Toward elucidating the role of number of oxazine rings and intermediates in the benzoxazine backbone on their thermal characteristics. Macromolecules.

[16] Kolanadiyil S.N., Azechi M., Endo T. (2016). Synthesis of novel tri-benzoxazine and effect of phenolic nucleophiles on its ring-opening polymerization. J. Polym. Sci. Part A Polym. Chem..

[17] Zhang K., Han M.C., Liu Y., Froimowicz P. (2019). Design and synthesis of bio-based high performance tri-oxazine benzoxazine resin via natural renewable resources. ACS Sustain. Chem. Eng..

[18] Zhang K., Han M.C., Han L., Ishida H. (2019). Resveratrol-based tri-functional benzoxazines: Synthesis, characterization, polymerization, and thermal and flame retardant properties. Eur. Polym. J..

[19] Wang D., Li B., Zhang Y., Lu Z. (2013). Triazine-containing benzoxazine and its high-performance polymer. J. Appl. Polym. Sci..

[20] Alhwaige A.A., Alhassan S.M., Katsiotis M.S., Ishida H., Qutubuddin S. (2015). Interactions, Morphology and Thermal Stability of Graphene-Oxide Reinforced Polymer Aerogel Derived from Star-Like Telechelic Aldehyde- Terminal Benzoxazine Resin. RSC Adv..

[21] Alhwaige A.A., Ishida H., Qutubuddin S. (2019). Poly(benzoxazine-f-chitosan) films: The role of aldehyde neighboring groups on chemical interaction of benzoxazine precursors with chitosan. Carbohyd. Polym..

[22] Cash J.J., Davis M.C., Ford M.D., Groshens T.J., Guenthner A.J., Harvey B.G., Lamison K.R., Mabry J.M., Meylemans H.A., Reams J.T. (2013). High Tg thermosetting resins from resveratrol. Polym. Chem..

[23] Vervandier-Fasseur D., Chalal M., Meunier P. (2013). Method for Producing Trans-Resveratrol and the Analogs Thereof. WO Patent.

[24] Subbaraju G.V., Mahesh M., Hindupur H.R., Suresh T., Ivanisevic I., Andres M., Stephens K. (2011). Key Intermediate for the Preparation of Stilbenes, Solid Forms of Pterostilbene, and Methods for Making the Same. U.S. Patent.

[25] Fan E., Zhang K., Zhu M., Wang Q. (2010). Obtaining resveratrol: From chemical synthesis to biotechnology production. Mini-Rev. Org. Chem..

[26] Tian Y., Wang Q., Shen L., Cui Z., Kou L., Cheng J., Zhang J. (2020). A renewable resveratrol-based epoxy resin with high Tg, excellent mechanical properties and low flammability. Chem. Eng. J..

[27] Sawaryn C., Landfester K., Taden A. (2010). Benzoxazine miniemulsions stabilized with polymerizable nonionic benzoxazine surfactants. Macromolecules.

[28] Dunkers J., Ishida H. (1995). Vibrational assignments of 3-alkyl-3, 4-dihydro-6-methyl-2H-1,3-benzoxazines in the Fingerprint Region. Spectrochim. Acta Part A.

[29] Han L., Iguchi D., Gil P., Heyl T.R., Sedwick V.M., Arza C.R., Ohashi S., Lacks D.J., Ishida H. (2017). Oxazine ring-related vibrational modes of benzoxazine monomers using fully aromatically substituted, deuterated, ^15^N isotope exchanged, and oxazine-ring-substituted compounds and theoretical calculations. J. Phys. Chem. A.

[30] Sini N.K., Azechi M., Endo T. (2015). Synthesis and properties of spiro-centered benzoxazines. Macromolecules.

[31] Zhang K., Shang Z., Evans C.J., Han L., Ishida H., Yang S. (2018). Benzoxazine atropisomers: Intrinsic atropisomerization mechanism and conversion to high performance thermosets. Macromolecules.

[32] Zhang K., Liu Y., Evans C.J., Yang S.F. (2020). Easily processable thermosets with outstanding performance via smart twisted small-molecule benzoxazines. Macromol. Rapid Commun..

[33] Wang J., Wu M., Liu W., Yang S., Bai J., Ding Q., Li Y. (2010). Synthesis, curing behavior and thermal properties of fluorine containing benzoxazines. Eur. Polym. J..

[34] Zhang K., Tan X.X., Wang Y.T., Ishida H. (2019). Unique self-catalyzed cationic ring-opening polymerization of a high performance deoxybenzoin-based 1,3-benzoxazine monomer. Polymer.

[35] Hamerton I., Thompson S., Howlin B.J., Stone C.A. (2013). New method to predict the thermal degradation behavior of polybenzoxazines from empirical data using structure property relationships. Macromolecules.

