# Supplementary material for: Design of High-Performance Polybenzoxazines with Tunable Extended Networks Based on Resveratrol and Allyl Functional Benzoxazine
Source: Polymers (Basel). 2020 Nov 26;12(12):2794. doi: 10.3390/polym12122794 (PMC7761256; doi:10.3390/polym12122794)
Supplement: Supplementary file 1 [file polymers-12-02794-s001.pdf]

polymers

Supporting Information for

Design of High-Performance Polybenzoxazines with Tunable Extended Networks Based on Resveratrol and Allyl Functional Benzoxazine

Yunliang Xing [1], Xianru He [1,*], Rui Yang [2], Kan Zhang [2,*], Shengfu Yang [3]

[1] School of New Energy and Materials, Southwest Petroleum University, Chengdu 610500, China; Phoebe@coryes.com (Y.X.)
[2] Research School of Polymeric Materials, School of Materials Science and Engineering, Jiangsu University, Zhenjiang 212013, China; yr183346@163.com (R.Y.); zhangkan@ujs.edu.cn (K.Z.)
[3] Department of Chemistry, University of Leicester, Leicester LE1 7RH, United Kingdom; sfy1@le.ac.uk (S.Y.)
* Correspondence: xrhe@swpu.edu.cn (X.H.);zhangkan@ujs.edu.cn (K. Z.); Tel.: +86-028-83037406 (X.H.); +86-0571-85016616 (K.Z.)

Received: 22 October 2020; Accepted: 24 November 2020; Published: date

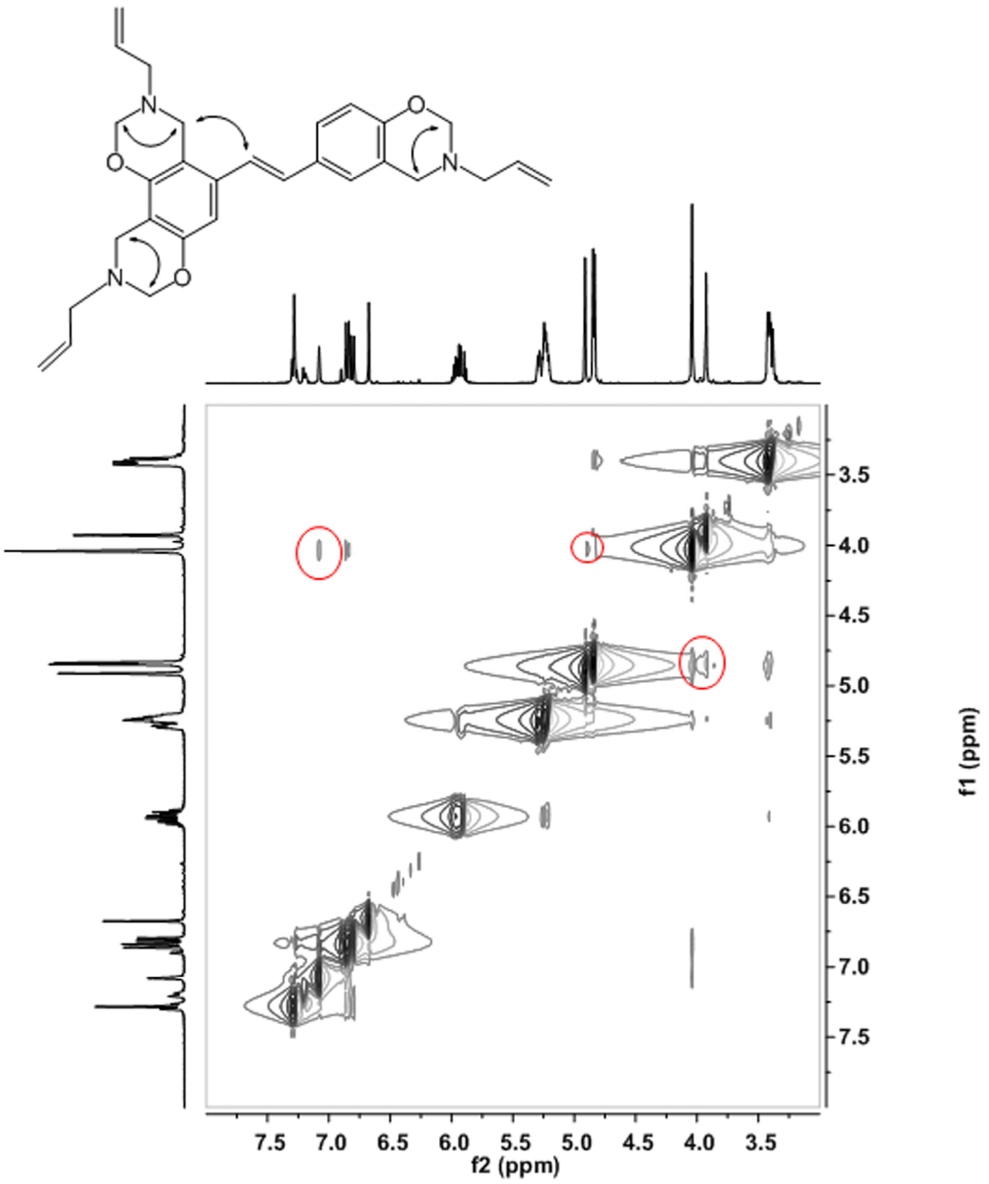

Figure S1. ^{1}H-^{1}H NOESY 2D NMR spectrum of RES-al.

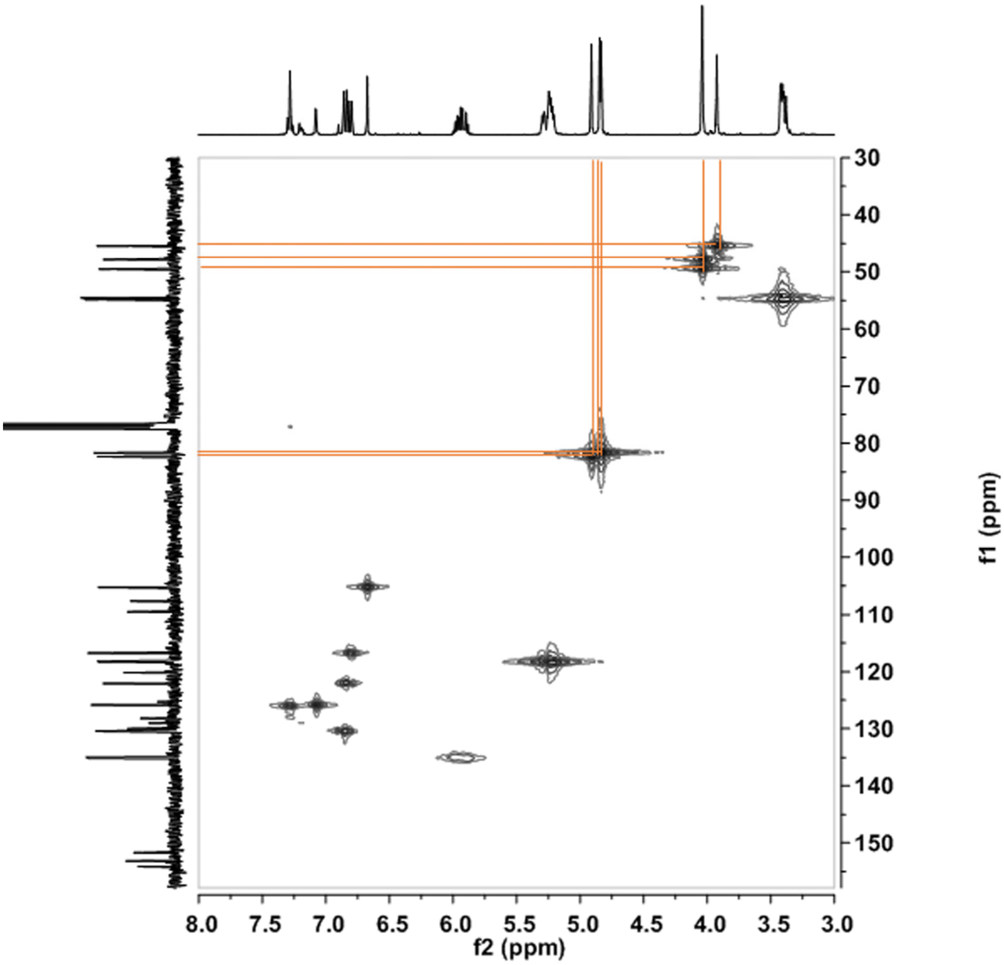

Figure S2. ^{1}H-^{13}C HMQC 2D NMR spectrum of RES-al.

© 2020 by the authors. Licensee MDPI, Basel, Switzerland. This article is an open access article distributed under the terms and conditions of the Creative Commons Attribution (CC BY) license (http://creativecommons.org/licenses/by/4.0/).
